# Selected Biochemical, Hematological, and Immunological Blood Parameters for the Identification of Malnutrition in Polish Senile Inpatients: A Cross-Sectional Study

**DOI:** 10.3390/jcm14051494

**Published:** 2025-02-23

**Authors:** Katarzyna Mądra-Gackowska, Karolina Szewczyk-Golec, Marcin Gackowski, Iga Hołyńska-Iwan, Dominika Parzych, Jolanta Czuczejko, Michał Graczyk, Jakub Husejko, Tomasz Jabłoński, Kornelia Kędziora-Kornatowska

**Affiliations:** 1 Department of Geriatrics, Faculty of Health Sciences, L. Rydygier Collegium Medicum in Bydgoszcz, Nicolaus Copernicus University in Torun, Skłodowskiej Curie 9 Street, PL–85094 Bydgoszcz, Poland; dominika.gebka@cm.umk.pl (D.P.); kubahusejko@gmail.com (J.H.); kornelia.kornatowska@cm.umk.pl (K.K.-K.); 2 Department of Medical Biology and Biochemistry, Faculty of Medicine, L. Rydygier Collegium Medicum in Bydgoszcz, Nicolaus Copernicus University in Torun, Karłowicza 24 Street, PL–85092 Bydgoszcz, Poland; karosz@cm.umk.pl; 3 Department of Toxicology and Bromatology, Faculty of Pharmacy, L. Rydygier Collegium Medicum in Bydgoszcz, Nicolaus Copernicus University in Torun, A. Jurasza 2 Street, PL–85089 Bydgoszcz, Poland; marcin.gackowski@cm.umk.pl; 4 Department of Pathobiochemistry and Clinical Chemistry, Faculty of Pharmacy, Ludwik Rydygier Collegium Medicum in Bydgoszcz, Nicolaus Copernicus University in Torun, Skłodowskiej Curie 9 Street, PL–85094 Bydgoszcz, Poland; igaholynska@cm.umk.pl; 5 Department of Psychiatry, Faculty of Medicine, L. Rydygier Collegium Medicum in Bydgoszcz, Nicolaus Copernicus University in Torun, Skłodowskiej Curie 9 Street, PL–85094 Bydgoszcz, Poland; joczu@cm.umk.pl; 6 Department of Palliative Care, Faculty of Health Sciences, L. Rydygier Collegium Medicum in Bydgoszcz, Nicolaus Copernicus University in Torun, Skłodowskiej Curie 9 Street, PL–85094 Bydgoszcz, Poland; michal.graczyk@cm.umk.pl; 7 Faculty of Health Sciences and Physical Culture, Kazimierz Wielki University, PL–85064 Bydgoszcz, Poland; tomas82@ukw.edu.pl

**Keywords:** malnutrition, the mini nutritional assessment (MNA), the geriatric nutrition risk index (GNRI), the global leadership initiative on malnutrition (GLIM), venous blood parameters, older adults, comprehensive geriatric assessment (CGA), interleukin 6, interferon γ-induced protein 10, melatonin

## Abstract

**Background/Objectives**: Malnutrition in senile patients leads to functional disability while reducing quality of life. Medical professionals should routinely assess their nutritional status during hospitalization. However, diagnosing malnutrition may be difficult, especially since obesity may mask malnourishment. Thus, it is essential to search for biomarkers that improve the identification of malnourished inpatients. **Methods**: In the present cross-sectional study, selected venous blood parameters were analyzed in 137 older inpatients at the age of 80.5 ± 7.78 admitted to the Geriatrics Clinic of the Antoni Jurasz University Hospital No. 1 in Bydgoszcz, Poland between 2017 and 2018, for a comprehensive geriatric assessment. The participants were grouped according to their nutritional risks based on the Mini Nutritional Assessment (MNA) and the Geriatric Nutrition Risk Index (GNRI). The Kruskal–Wallis test was utilized to evaluate the equality of variances for a variable calculated for two or more groups. The level of significance was set at *p* < 0.05. **Results**: For total protein, albumin, homocysteine, hemoglobin, hematocrit, total magnesium, total calcium, C-reactive protein (CRP), interleukin 6 (IL-6), and interferon γ-induced protein 10 (IP-10), statistically significant differences were found between groups of patients classified by the MNA. However, additional significant differences were also observed for creatinine, folic acid, and triglycerides, according to the GNRI compartmentalization. The results indicate that decreased levels of albumin (<3 g/dL) and hemoglobin (<11 g/dL), along with elevated homocysteine, CRP, IL-6 (>7.5 pg/mL), and IP-10 (>250 pg/mL), should alert medical professionals to potential malnutrition in hospitalized patients. **Conclusions**: Routine analysis of venous blood parameters can help rapidly identify malnutrition and the immediate implementation of a specialized diet.

## 1. Introduction

Malnutrition has been a topic of significant scientific interest in recent decades and is now considered one of the geriatric giants [1]. It can lead to serious consequences such as depression and sarcopenia, which can cause functional disability [2]. As people age, their appetite regulation system changes, which can lead to the so-called anorexia of aging. It predisposes older adults to a decrease in food intake, which can lead to malnutrition when combined with additional risk factors like health or social problems. This condition is widespread, especially among institutionalized older adults. On the other hand, obesity has become a significant problem in the geriatric population worldwide [3]. Obesity increases the risk of other diseases, but it could also mask the presence of malnutrition [4]. The term “geriatric giants” refers to chronic disabilities associated with aging that can lead to adverse health outcomes. Patients, caregivers, and even physicians may consider these conditions to be a natural consequence of aging, and they often do not receive enough attention until they impose a significant burden on patients and the healthcare system [1,2,5]. However, assessing nutritional status is essential to a comprehensive geriatric assessment (CGA) [6,7]. Although effective nutritional interventions are available, prevention and treatment of malnutrition do not currently receive appropriate attention.

Malnutrition is a deficiency, excess, or imbalance of essential nutrients reflected in the patient’s clinical and body condition and affects vital functions. In the case of older patients, the most common deficiencies are protein and energy [8]. As a condition that results from starvation, disease, or aging alone or in combination, malnutrition can also be defined as “a state resulting from lack of uptake or intake of nutrition leading to altered body composition (decreased fat-free mass) and body cell mass leading to diminished physical and mental function and impaired clinical outcome from disease” [9]. This definition is well accepted, but till 2018, there were no generally accepted diagnostic criteria. In 2018, the Global Leadership Initiative on Malnutrition (GLIM) proposed a two-step approach to malnutrition diagnosis. The first step is a screening to identify “at risk” status using any validated screening tool, and the second is an assessment for diagnosis and grading the severity of malnutrition [10]. Malnutrition is a severe medical issue among senile people that directly affects their hospital stay duration, medical expenses, infection frequency, complications, and mortality rate [11]. Malnourished individuals experience a decline in their functional capacity and life quality. Malnutrition can also lead to walking difficulties, falls, and fractures. The root causes of poor nutritional status in older patients are multifaceted, including physiological, psychological, and social changes that result in reduced food intake and decreased body weight [12]. After hospitalization, the nutritional status of older patients usually worsens due to increased nutrient requirements, nausea, loss of appetite, aversion to certain foods, or difficulties with oral intake. Despite the high prevalence of malnutrition among hospitalized individuals and the availability of recognized methods to identify malnourished patients, screening is often not performed, and malnutrition is rarely identified in healthcare facilities [13]. The prevalence of malnutrition in older hospitalized patients varies from 30% to 90%, depending on the identification method used [11].

Malnutrition is caused by various factors, including aging, diseases, medications, and the environment [5]. With age, the rate of basal metabolism and total energy expenditure decreases, saliva secretion is impaired, taste bud atrophy, and senses of smell and taste are affected, all of which increase the risk of malnutrition. Diseases such as cancer, heart failure, chronic obstructive pulmonary disease, chronic kidney disease, hyperthyroidism, depression, stroke, Parkinson’s disease, dementia, and other conditions that cause dysphagia, nausea, vomiting, diarrhea, or constipation can also lead to malnutrition [14]. Pharmacotherapy can affect appetite, hunger, satiety, and nausea, impacting the amount of food consumed. Environmental factors such as lack of dentures, unattractive meal presentation, and unfavorable socioeconomic conditions can also contribute to malnutrition [5,14].

Changes in body composition are characteristic of the aging process. There is a gradual increase in visceral adipose tissue and a decrease in lean body mass [15]. Moreover, the amount of water content, mainly intracellular water, also declines, decreasing the amount of potassium in the senior body. This water loss can be significant, especially in women between the third and eighth decades of life, up to 17%, and in men, up to 11%. Older people also experience a decrease in skeletal muscle mass, and women in menopause are at a higher risk of developing osteoporosis due to a progressive reduction in bone density, which also increases the risk of fractures [8,15]. All these changes in body composition lead to changes in seniors’ nutritional needs and requirements. A decrease in skeletal muscle mass reduces the rate of basal metabolism, and a reduction in food consumption decreases diet-induced thermogenesis, and physical activity often declines, leading to a decrease in energy requirements of up to 20% [8]. The above changes result in a positive energy balance and increased body weight. However, at a later age, due to anorexia nervosa, the energy balance shifts to negative, resulting in a decrease in the values of Body Mass Index (BMI) and fat mass [15].

The MNA is a widely used and recommended tool for assessing malnutrition in older adults, especially in clinics, hospitals, and nursing homes, according to the European Society for Clinical Nutrition and Metabolism (ESPEN) [16]. Primary care physicians can also use it to quickly diagnose malnutrition in patients who need a more extensive nutritional assessment. This tool includes physical and psychological aspects that affect the nutritional status of senile patients, along with a dietary questionnaire. That is why it is handy in seniors with frailty syndrome, as it identifies the risk of malnutrition and malnutrition itself at an early stage [17]. The Mini-Nutritional Assessment is the most common and effective tool used in seniors, and it can assess the risk of malnutrition before clinical changes occur [18,19]. The main limitation of this questionnaire is its relatively long interview time, which is about 10–15 min. To overcome this limitation, shorter versions of the MNA have been proposed, such as the MNA-SF (shortened version of the MNA), which contains only six questions. However, for a more in-depth assessment of the nutritional status of patients at risk of malnutrition, it is suggested that the full version of the MNA be used [16,18]. The Geriatric Nutritional Risk Index is a valuable tool for evaluating the nutritional status of older adults [20]. It is a modified version of the Nutritional Risk Index (NRI), which is not very helpful for seniors as it includes body weight, which most older people cannot control or remember [21]. Instead, the GNRI calculates the ideal body weight using the Lorentz equation. Although the GNRI is not the gold standard in assessing malnutrition risk, it can effectively enhance nutritional status evaluation with other parameters. The index is designed for patients aged 65 and above to identify and predict potential nutritional-related complications [21]. It has also been suggested that malnutrition is linked to poor health, so the GNRI can help identify patients who need nutritional treatment. The GNRI is helpful when the MNA cannot be used or as a supplement to the MNA questionnaire for hospitalized patients [22].

Biochemical markers can help assess nutritional status, but laboratory results cannot solely confirm with complete certainty whether a patient is malnourished. These markers are not considered screening tools due to their lack of specificity, though they can complement diagnostics. When deviations from typical values are observed, it is difficult to determine whether they are related to the aging process, the disease process, medications and supplements, or malnutrition. Standard laboratory tests for assessing malnutrition in senile patients include total lymphocyte count, albumin concentration, cholesterol level, hemoglobin level, transferrin level, and hematocrit [23]. Inflammation, often associated with disease or aging, is probably the most common cause of malnutrition [24]. Therefore, inflammation is an essential factor in the development of malnutrition, as indicated by markers such as albumin, prealbumin, and C-reactive protein (CRP). The serum albumin concentration is not only the most widely used marker of nutritional status but also reflects the negative impact of malnutrition on protein synthesis [25]. A decrease in albumin concentration can also occur in patients due to ongoing disease processes, inflammatory processes, wounds, and edema.

Human aging is inextricably linked to changes in the immune system. One such change is a chronic subthreshold inflammatory state characterized by increased inflammatory cytokines circulating in the body, which can be 2–4 times higher than usual. Among these cytokines, interleukin 6 (IL-6) is particularly important for gerontologists and is called the “gerontologist’s cytokine”. IL-6 has pro- and anti-inflammatory effects depending on the concentration, timing, and location of its activity on cells. Its concentration in the blood increases with age and is higher in patients with sarcopenia and frailty syndrome [26]. Recently, there has been a growing interest in interferon γ-induced protein 10 (IP-10), a pro-inflammatory chemokine that also serves as a marker of oxidative stress. Cross-sectional studies have indicated that IP-10 levels, IL-6, interleukin 18 (IL-18), and adiponectin, are linked to insulin resistance [27,28]. Melatonin is a hormone secreted by the pineal gland. It has various effects, such as regulating circadian cycles and energy metabolism. It also plays a role in reducing oxidative stress as an antioxidant and in regulating immune function. Additionally, melatonin has been found to normalize the expression and secretion of two major adipokines: leptin and adiponectin [29].

The seriousness of the malnutrition problem is emphasized by the results of the PolSenior study conducted in the Polish senile population in the first decade of the 21st century, where the MNA was used to assess the nutritional status of the study participants. The prevalence of malnutrition was 7.5%, and the estimated risk of malnutrition may affect up to 40% of the population [30]. In general, malnutrition is 3.10% among older people in the community, while it rises to 22.00% among hospitalized older adults [31].

Considering the above scientific reports demonstrating the nature and relatively high prevalence of malnutrition in the senile population worldwide, the current study aimed to shed light and give details on the incidence of malnutrition in the second decade of the 21st century among older patients in central and eastern Europe, using Poland as an example, who were referred by a general practitioner to hospital for the CGA. As the diagnosis of malnutrition is challenging and this problem is often overlooked in clinical practice, this study primarily focused on searching for biomarkers that may facilitate and accelerate the identification of malnourished individuals. This approach aimed to explore the links between selected venous blood parameters and the nutritional risks based on the MNA and the GNRI, taking into consideration routinely tested blood indices during hospitalization and such parameters as melatonin, IL-6, and IP-10.

## 2. Materials and Methods

### 2.1. Study Participants

For the current cross-sectional observational study, 137 senile patients (96 women and 41 men) who were referred by the family doctor to the Geriatrics Clinic of the Antoni Jurasz University Hospital No. 1 in Bydgoszcz, Poland, for a comprehensive geriatric assessment were recruited on the admittance to the clinic by the attending physician (Patients were asked whether they would like to participate in this single-center cross-sectional study or not). The present study was conducted from June 2017 to December 2018. The average age of the patients was 80.5 years, with a standard deviation of approximately 7.78. The study received ethical approval from the Bioethics Committee of Ludwik Rydygier Collegium Medicum in Bydgoszcz, Nicolaus Copernicus University in Toruń, Poland (Consent No. KB 134/2016). All participants were fully informed about the study’s purpose and principles and provided written consent to participate. In cases of moderate dementia, relatives or guardians gave written consent for patients to participate in the study. The inclusion criteria were being 65 or older and in adequate physical condition to undergo a complete examination. Patients who were unable to move independently, bedridden, diagnosed with Parkinson’s disease, cancer, or acute illness, and had deep cognitive disorders that prevented full contact with the patient (Mini-Mental State Examination (MMSE) score less than 10) were excluded from the study.

### 2.2. Data Collection

All participants underwent a comprehensive geriatric assessment (CGA). The objective was to assess each individual’s nutritional, physical functioning, psychological, and social status globally. The assessments were performed by a multi-disciplinary team composed of physicians, physiotherapists, nutritionists, psychologists, and nurses. Face-to-face interviews were conducted with 137 senile people who agreed to participate in the study. Data on gender, age, educational level, medical history, and feeding habits were collected using a standardized questionnaire. Moreover, the full Mini Nutritional Assessment (MNA) questionnaire was used for malnutrition screening. Interviews were conducted by qualified medical professionals.

The MNA questionnaire [17] was used in the first step of the evaluation of the nutritional status of hospitalized seniors as a malnutrition risk screening tool. This tool assesses the current nutritional status of each participant and classifies individuals into one of three categories: malnutrition, risk of malnutrition, or well-nourished. The classification is based on a scoring system, where scores below 17 out of 30 indicate “malnutrition”, scores from 17 to 23.5 indicate “at risk of malnutrition”, and scores above 23.5 indicate “normal nutritional status”. This tool is available in several languages and serves as a reliable predictor of mortality and hospital costs associated with malnutrition. The second step was a diagnosis of malnutrition and its severity grading according to the GLIM criteria [10]. Additionally, the Geriatric Nutritional Risk Index [32] was used as a complementary tool to the MNA questionnaire. 

The research involved collecting blood samples to determine biochemical and hematological parameters of venous blood (total protein, albumin, homocysteine, hemoglobin, hematocrit, total magnesium, total calcium, creatinine, vitamin D3, vitamin B12, folic acid, WBC, C-reactive protein, cholesterol, HDL cholesterol, LDL cholesterol, and triglycerides), routinely tested during a hospital stay due to the CGA. Qualified medical professionals collected blood samples for biochemical tests in the morning (between 8:00 a.m. and 9:00 a.m.) after overnight fasting from the cubital vein into two polypropylene tubes. Two tubes (with a volume of 4 mL each) with a clotting activator were used to obtain blood serum, and another tube (with a volume of 4 mL) containing K_2_EDTA allowed the preparation of blood plasma and erythrocytes. Melatonin concentration was determined in blood plasma using the Enzyme-linked Immunosorbent Assay Kit For Melatonin (Cloud-Clone Corp., Katy, TX, USA). The assay used a competitive ELISA to directly measure melatonin in human serum, plasma (collected on EDTA, heparin), and other body fluids. The concentration of IL-6 was determined in serum using the Human IL-6 High Sensitivity ELISA Kit (DIACLONE SAS, Besançon, France). The assay used was a double-binding sandwich ELISA designed to measure interleukin 6 in human serum and plasma directly. IP-10 concentration was determined in serum using the Human IP-10 ELISA Kit (DIACLONE SAS, Besançon, France). The assay used was a double-binding sandwich ELISA designed to directly measure interferon γ-induced 10 kDa protein in human serum and plasma.

### 2.3. Statistical Analysis

The statistical analysis of the results was carried out using Statistica 13.3 (TIBCO Software Inc., Palo Alto, CA, USA). Pearson correlation analysis was conducted to test the degree of association between nutritional status determined by the MNA and malnutrition risk according to the GNRI. The Shapiro–Wilk test was conducted to test the hypothesis of normal distribution. For independent samples, the Student’s t-test was used. However, the requirements for a one-factor analysis of variance were not met, and therefore, the Kruskal–Wallis test was utilized as a nonparametric alternative to evaluate the equality of variances for a variable calculated for two or more groups. The results are presented in the form of tables and charts. The level of significance was set at *p* < 0.05.

## 3. Results

According to the MNA questionnaire, among 137 recruited senile inpatients, 47 were malnourished, 12 were at risk of malnutrition, and 78 had a normal nutritional status. It corresponds to 34.31%, 8.76%, and 56.93% of the studied population, respectively (Table 1). Malnutrition diagnosis according to the GLIM consensus [10], based on the presence of at least one phenotypic criterion (non-volitional weight loss, low BMI, or/and reduced muscle mass), and one etiologic criterion (reduced food intake or assimilation, and inflammation or disease burden), confirmed malnutrition in all patients indicated by the MNA screening tool. Considering phenotypic criteria for grading severity of malnutrition, 35 patients (74.47%) suffered from moderate and 12 individuals (25.53%) from severe malnutrition. Therefore, it was found that a significant number of individuals in the studied population were undernourished.

This was confirmed by the GNRI classification, which identified 50 patients at risk of nutritional-related complications. Of these, 12 were at high risk, 17 at moderate risk, and 21 at low risk, corresponding to 8.76%, 12.41%, and 15.33%, respectively. However, 63.50% of study participants were not at risk of any nutritional-related complications. Based on the MNA score, the patients were divided into three groups: those with adequate nutritional status, those at risk of malnutrition, and those malnourished. Additional division, according to the GNRI, was also taken into account. It helped identify four groups of patients varying in degree of risk of nutritional-related complications: major risk (<82), moderate risk (82–<92), low risk (92–≤98), and no risk (>98).

### 3.1. Correlation Between the MNA and the GNRI

**Figure 1:**
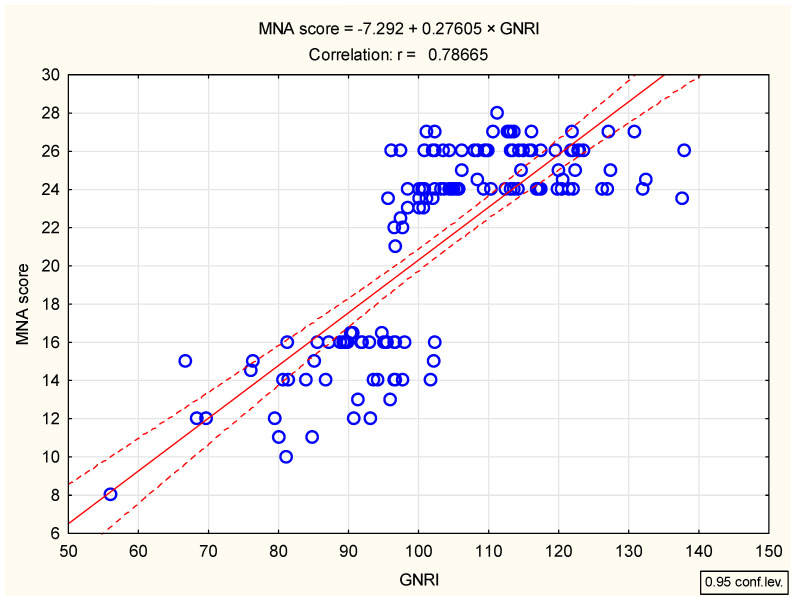
2W-scatter plot for the MNA scores versus the GNRI values.

In the present cross-sectional study conducted, the degree of association between nutritional status as determined by the MNA and the risk of nutritional-related complications according to the GNRI was examined. For this purpose, a thorough Pearson correlation analysis was performed (Table 2) and a 2W-scatter plot was created (Figure 1).

A powerful relationship was found between the MNA and the GNRI in determining the nutritional status of patients, with a correlation coefficient of approximately 79%. The coefficient of determination shows that around 62% of the MNA variables were determined by regression versus the GNRI. Although both the MNA and the GNRI are highly correlated, there are differences due to different measurement methods. However, both indicators determine the nutritional status of study participants in a similar way.

### 3.2. Selected Parameters of Venous Blood Versus Nutritional Status Based on the MNA

In this stage of the study, selected parameters of venous blood were measured, and the obtained data were compared between different groups based on the MNA. The Kruskal–Wallis test was utilized for statistical inference, and the estimation was made at a significance level of α = 0.05 (Table 3). Variables that showed significant differences are highlighted in blue.

**Figure 2:**
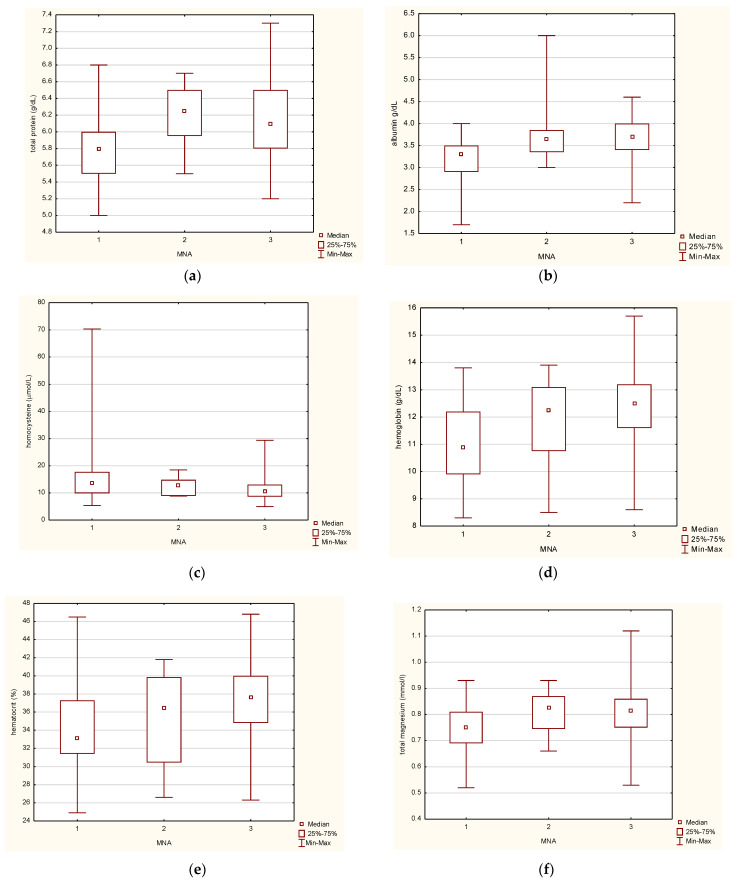

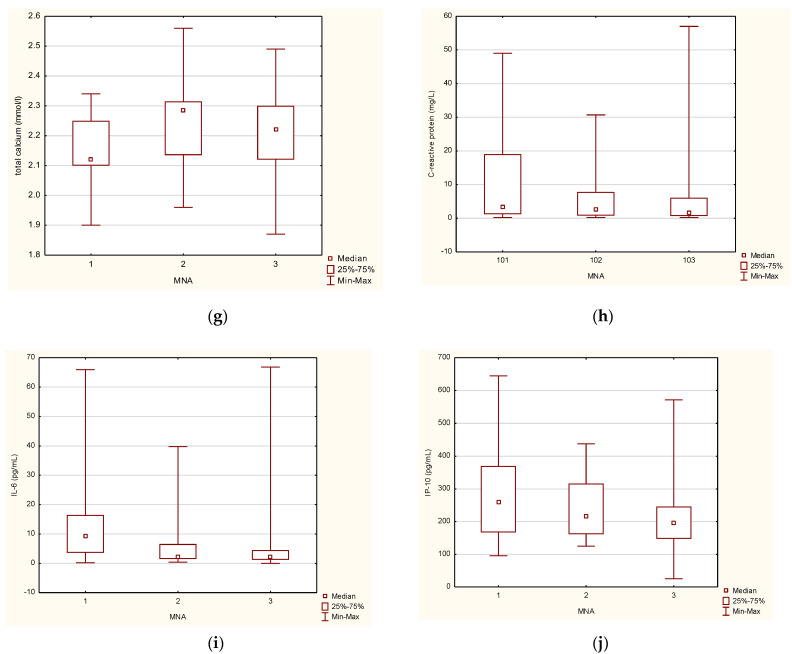
Boxplots for selected venous blood parameters in the intergroup comparison based on the MNA (1—malnutrition; 2—at risk of malnutrition, 3—normal nutritional status 4): total protein (**a**); albumin (**b**); homocysteine (**c**); hemoglobin (**d**); hematocrit (**e**); total magnesium (**f**); total calcium (**g**), C-reactive protein (**h**), interleukin 6 (**i**), and interferon γ-induced protein 10 (**j**).

The study confirmed the differences between the groups determined by the MNA for the following variables: total protein, albumin, homocysteine, hemoglobin, hematocrit (HCT), total magnesium, total calcium, C-reactive protein, IL-6, and IP-10. Further, the experimental data were interpreted based on the box plots (Figure 2).

Testing of remaining studied parameters, including creatinine, vitamin D3, vitamin B12, folic acid, white blood cells (WBC), total cholesterol, high-density lipoprotein (HDL) cholesterol, low-density lipoprotein (LDL) cholesterol, triglycerides, and melatonin did not show any significant differences between groups. However, there was a slight indication of significance in the variables triglycerides and cholesterol, but the established significance level of α = 5% excluded these results (Table 3).

The analysis of the total protein levels in the blood of older individuals showed some notable differences. Based on the boxplot findings (Figure 2a), there appears to be a reduction in total protein levels in the blood of those who were malnourished, as determined by the MNA. Similarly, the analysis of albumin levels in the blood showed a relationship with malnutrition. The study group of malnourished individuals selected using the MNA questionnaire tended to lower albumin concentrations (Figure 2b). While the median albumin levels from the groups of inpatients with at-risk malnutrition and normal nutritional status were similar, the range of values suggested that the number of people with high albumin concentrations was higher in the group with normal nutritional status. Finally, the amino acid homocysteine levels were found to differ significantly between the groups of people classified as malnourished and those with normal nutritional status. In the former group, the homocysteine concentration in the blood was markedly higher (Figure 2c). The study also showed variability in the hemoglobin (Hb) being studied. Based on the results presented in Figure 2d, it can be inferred that Hb concentrations were lowest in the malnourished cohort. As the nutritional status improved, the median amount of this parameter increased sequentially in the studied groups: those at risk of malnutrition and those with normal nutritional status. The blood hematocrit also differed among the study groups. The most significant median differences were observed between the malnutrition and normal nutritional status groups despite a similar spread of data (Figure 2e) demonstrating that there were more subjects with low HCT in the malnourished group. Blood magnesium levels were found to increase in patients with normal nutritional status compared to those with malnutrition (Figure 2f). Though the increase was insignificant, it differed significantly when comparing the two groups. An intriguing finding of the analysis was that the group of people with malnutrition significantly differed from the group at risk of malnutrition in terms of the total calcium factor studied (Figure 2g). It was observed that an increase in calcium levels was noted among individuals who had normal nutritional status when compared to those who were malnourished. However, the specificity of these results differs from the previous analyses. Furthermore, the analysis for C-reactive protein showed statistically significant differences between the two groups, as depicted in Figure 2h. The median values for each of the three groups were found to be at a similar level. Hence, it is not possible to differentiate between the groups. A study with a larger number of cases is required to determine differences between the groups. In the case of IL-6, the highest levels were found in malnourished subjects, while lower concentrations were found in normally nourished patients (Figure 2i). In the group of subjects at risk of malnutrition, the sample median is shifted toward lower Il-6 concentrations, associated with the subjects’ normal nutritional status. IP-10 is also a factor for detecting differences relative to the MNA (Figure 2j). Analogous to Il-6, a decrease in its levels negatively correlates with improved nutritional status. The lowest median was determined for the patients with normal nutritional status. In addition, it differs, at a statistically significant level, from the group of malnourished patients, which makes it possible to distinguish these groups.

### 3.3. Selected Parameters of Venous Blood Versus Nutritional Status Based on the GNRI

As a part of statistical analysis, it was examined whether there were any significant differences in the levels of venous blood parameters among the groups of inpatients determined by the GNRI. After a preliminary analysis using the Kruskal–Wallis, test key variables that showed significant differences were identified. These variables are highlighted in blue in Table 4.

**Figure 3:**
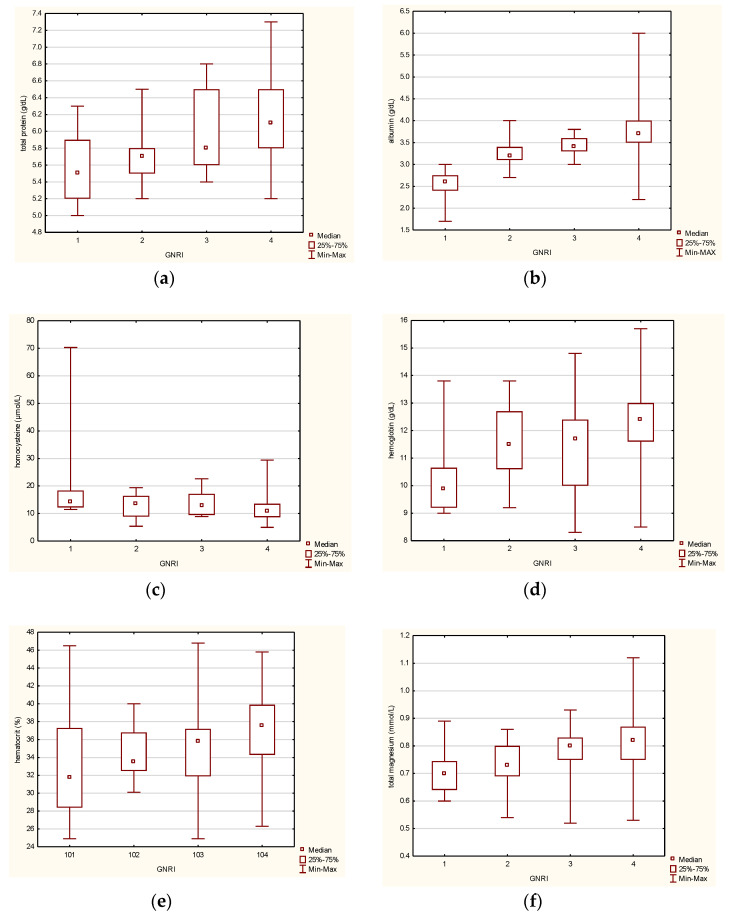

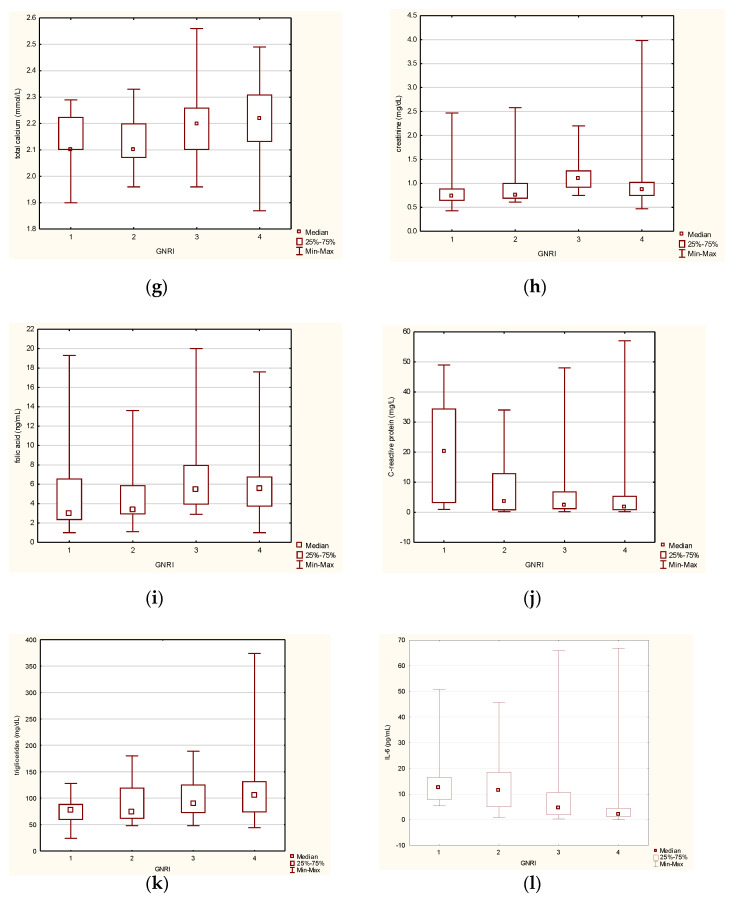
Boxplots for selected venous blood parameters in the intergroup comparison based on the GNRI (1—high risk, 2—medium risk, 3—low risk, 4—no risk of nutritional-related complications): total protein (**a**); albumin (**b**); homocysteine (**c**); hemoglobin (**d**); hematocrit (**e**); total magnesium (**f**); total calcium (**g**), creatinine (**h**), folic acid (**i**), C-reactive protein (**j**), triglycerides (**k**), and interleukin 6 (**l**).

The results of a study on selected parameters of venous blood showed that some of the tested indices have a specific variability concerning nutritional-related risk complications determined by the GNRI. These variables include total protein, albumin, Hb, homocysteine, HCT, magnesium, calcium, C-reactive protein, and IL-6. These parameters were also significant for the nutritional status based on the MNA. However, in the case of the GNRI compartmentalization, group variability was also observed for the variables creatinine, folic acid, and triglycerides. Further analysis was conducted based on the box plots presented in Figure 3 to interpret the experimental data appropriately. These findings demonstrate the data variability between the compared groups, and statistically significant results were achieved.

Testing parameters such as vitamin D3, vitamin B12, WBC, total cholesterol, HDL cholesterol, LDL cholesterol, melatonin, and IP-10 did not yield the expected results. Consequently, these variables were excluded from further analysis.

The result obtained from the analysis of the total protein concerning nutritional-related complications risk, expressed as the GNRI index value, is very similar to that obtained for the MNA grouping variable (Figure 3a). The amount of total protein in the group with no risk of nutritional-related complications is significantly different and higher than in the fraction with high risk. Based on the median values in Figure 3a, it can be inferred that the total protein concentration found in the blood increases with a decrease in the risk of malnutrition. Relative to the GNRI grouping variable, albumin concentrations increase as the risk of malnutrition decreases. Figure 3b illustrates the highest albumin concentration in the no-malnutrition risk group. The subjects’ blood albumin concentration is the lowest for the highest nutritional-related complications risk. The tested homocysteine level in the recruited inpatients is highest in those at high risk of malnutrition (Figure 3c). A decreasing level of this parameter was observed as the risk of malnutrition decreased. This result correlates with the one previously obtained for the MNA grouping variable. The hemoglobin level showed a decrease as the risk of nutritional-related complications increased. Figure 3d shows that the difference at a statistically significant level was observed between the groups with no risk and high risk of complications related to nutritional status. Adequate correlations were detected for the MNA questionnaire. The hematocrit analyzed and compared according to the GNRI (Figure 3e) also showed a similar relationship to the MNA grouping. The group with no risk of malnutrition had the highest median of HCT, while the group at high risk of malnutrition had the lowest median. The present study found that subjects with a higher magnesium concentration in their blood had a lower risk of malnutrition. The group with the lowest risk of poor nutritional status had the highest level of magnesium, whereas those at risk of malnutrition had lower levels (Figure 3f). Similar correlations were found for total calcium, with the group at no risk of malnutrition having the highest levels and the group at high or moderate risk having the lowest (Figure 3g). There were no differences in creatinine levels based on the MNA classification, but differences were found when grouping by GNRI. The no-risk group had a smaller median creatinine level than the low-risk group, but the whiskers in Figure 3h show that those with the highest creatinine levels were also in the no-risk group. However, there were too few cases in the study population to distinguish the group of well-nourished patients by their creatinine levels. Regarding the nutritional-related risk complications expressed by the GNRI, it was found that there were statistically significant differences between the low-risk and the moderate-risk groups for folic acid. However, additional analyses are required as the results remain inconclusive. The boxplot presented in Figure 3i shows less variation between the extreme groups. Using statistical techniques, the differentiation of C-reactive protein made it possible to distinguish the high-risk group from the low-risk and no-risk groups in terms of the concentration of this protein (Figure 3j). The median concentration of the protein in the first fraction is significantly higher. The protein concentration associated with inflammation decreases as the risk of malnutrition declines. However, these relationships were not shown at a statistically significant level for the MNA questionnaire. Finally, it was found that an increase in triglycerides was associated with a decrease in the risk of malnutrition. The low-risk and no-risk groups determined using the GNRI showed higher triglyceride concentrations compared to the high-risk and moderate-risk groups (Figure 3k). However, such relationships also were not confirmed using the MNA. IL-6 also belongs to the parameters for which significant differences were identified. The most extensive group divergence was determined for the fractions of high-risk and no risk of nutritional-related complications (Figure 3l). No risk of malnutrition determines the lowest median in the study population. Worsening of the nutritional status determines an increase in the amount of IL-6 in the blood of the studied patients.

## 4. Discussion

### 4.1. Prevalence and Significance of Malnutrition in Older Patients

Malnutrition in seniors, a significant issue often underestimated, is one of the Geriatric Giants that can lead to functional disability, mobility issues, incapacity, and reduced quality of life [5]. Shockingly, up to a quarter of patients at nutritional risk do not receive proper support or counseling from health professionals [33]. This underscores the urgent need to identify malnourished patients and implement appropriate nutritional management through screening tools for malnutrition and the search for new markers of malnutrition. While several studies have analyzed factors associated with malnutrition, their cross-sectional nature does not allow for a causal relationship to be established [34].

In the PolSenior study, as many as 7.5% of older malnourished persons aged 65 and over were identified in the Polish population, and the risk of malnutrition was found in almost 40% of the subjects [30]. The cited data are only the tip of the iceberg, and there is no accurate knowledge of the actual spread of the phenomenon. This highlights the need for further research to fully understand the prevalence of malnutrition. It is important to note that the prevalence of malnutrition in older inpatients can range from 30% to 90%, depending on the tool used to identify the nutritional status [11]. Every older patient admitted to the hospital must be screened for malnutrition. Such a diagnosis should be an integral part of the CGA. The present study conducted on 137 patients admitted to the Geriatrics Clinic of the Antoni Jurasz University Hospital No. 1 in Bydgoszcz, Poland, revealed that as much as 34.31% of patients were malnourished, and 8.76% were at risk of malnutrition according to the MNA questionnaire. The GNRI classification identified 50 patients at risk of nutritional-related complications, ranging from low to high risk. Out of these, 12 patients were at high risk, 17 were at moderate risk, and 21 were at low risk. It translates to 8.76%, 12.41%, and 15.33%, respectively. These statistics highlight the magnitude of the problem of malnutrition in older inpatients in central and eastern Europe, in the example of Poland. Practically every third patient admitted to the hospital in Poland may suffer from malnutrition, which should be considered during hospitalization for diagnosis, pharmacotherapy, or issuing adequate nutritional recommendations.

Malnutrition among senile hospitalized patients may be influenced by various factors, resulting in significant differences when assessed globally. A study conducted by Nazan et al. in a health center in the Afyonkarahisar province of Turkey found a similar number of malnourished individuals, i.e., 38.2% of the 102 patients assessed using the MNA were malnourished, 18.6% were at risk of malnutrition, and 43.1% had normal nutritional status [35]. On the other hand, another cross-sectional study conducted in Saudi Arabia identified 76.6% of hospitalized patients as malnourished or at risk of malnutrition. The study recruited 248 patients with a mean age of 70.0 ± 7.7 years, and nutritional status was assessed using the MNA-SF [36]. The abovementioned findings are consistent with the present investigation, underscore the need for better patient care, and emphasize that malnutrition is a widespread issue among older hospitalized patients, significantly associated with more extended hospital stays and increased mortality rates.

Malnutrition can lead to functional disability and mobility issues, especially in older adults often suffering from concomitant diseases such as osteoarthritis [37]. Patients suffering, for instance, from knee osteoarthritis experience pain, decreased joint mobility, stiffness, and muscle weakness, and long-term effects can lead to sleep disturbances, depression, and disability. In this light, not only are nutritional and pharmacological approaches necessary, but, first of all, non-invasive, pain-relieving, low side-effects, and cost-effective approaches are essential for these patients. As a physical factor therapy, therapeutic ultrasound is widely used in treating muscle and skeletal diseases with the advantages of non-invasiveness, convenience, and safety. It is also superior to promote physical activity among older patients for the prevention of mental health complications, which often accompany malnutrition in older individuals. Rahmati et al. [38] revealed a significant association between higher physical activity levels and reduced risk of depression, which was consistent across various age groups, sexes, and geographical regions.

Malnutrition is a problem that is often overlooked in older hospitalized patients. One way to identify this issue is by analyzing biochemical and hematological markers obtained during laboratory tests of blood samples taken from patients in the hospital. A systematic review conducted by Zhang et al. [39] examined 936 publications and 111 studies and identified 43 biomarkers present in the blood that can be used to identify malnourished patients. However, only 17 of these biomarkers were present in three or more studies. The most commonly studied biomarkers were albumin, hemoglobin, total cholesterol, total lymphocyte count, prealbumin, C-reactive protein, total protein, transferrin, creatinine, triglycerides, white blood cells, blood urea nitrogen, hematocrit, iron, and glomerular filtration rate. As part of a comprehensive geriatric evaluation in the present cross-sectional study, the biochemical and hematological parameters of venous blood were determined in patients. The study included the following parameters: hemoglobin, hematocrit, WBC, albumin, total protein, creatinine, homocysteine, vitamins (B12, D3, and folic acid), magnesium, calcium, lipid profile (triglycerides, total cholesterol, LDL cholesterol, and HDL cholesterol), and CRP. Despite the study’s limited size and its cross-sectional nature, it enabled the identification of hematological and biochemical indicators that change with nutritional status, as characterized by the MNA and the GNRI. These findings have practical implications for the identification and management of malnutrition in older adults.

### 4.2. The MNA and the GNRI for the Identification of Malnourished Inpatients

The MNA questionnaire is a widely used tool for assessing patients’ nutritional status, and the GNRI is often used in conjunction with it to identify malnourished geriatric patients [39]. The Geriatric Malnutrition Risk Index is a promising tool for predicting nutrition-related complications in hospitalized patients. In the present contribution, the conducted comparison between the MNA and the GNRI showed a very high correlation, with a coefficient of 79%, indicating a strong relationship between the variables (Figure 1). The coefficient of determination revealed that around 62% of the MNA variability was determined by regression of the GNRI variable. While the MNA questionnaire and the GNRI are used to assess nutritional status, they differ in their approaches, leading to discrepancies that do not allow for a more than 90–100% correlation. In a different study conducted on 134 senile patients (aged 68.9 ± 8.4 years) who were admitted to the intensive care units at Tuanku Ampuan Rahimah Hospital in Kelang, Malaysia, the Geriatric Nutritional Risk Index was compared with the Mini Nutritional Assessment using the Subjective Global Assessment (SGA) as a reference method [20]. The results showed that based on the SGA, the MNA, and the GNRI, 26.9%, 42.5%, and 44.0% of the participants were identified as malnourished, respectively. Similarly, the study concluded that the GNRI is a reliable tool and is comparable to the MNA in assessing the nutritional status of older hospitalized patients.

### 4.3. Venous Blood Versus Nutritional Status Based on the MNA

In the present cross-sectional study, statistical analysis confirmed differences in biochemical and hematological parameters between groups determined by the MNA for total protein, albumin, homocysteine, hemoglobin, hematocrit, total magnesium, total calcium, and CRP in the study population (Table 3). However, the expected divergence between groups was not shown for the other biomarkers. A reduction in blood proteins could be inferred in the group characterized by poor nutritional status (Figure 2a). Moreover, the group of malnourished subjects selected using the MNA showed a trend toward a decrease in blood albumin levels (Figure 2b). Although the medians in the groups with malnutrition risk and normal nutritional status were at a similar level, based on the spread of values, it can be concluded that the number of subjects with higher albumin levels was higher in the group with adequate nutritional status. The studied malnourished and well-nourished patients significantly differ in the tested level of the amino acid homocysteine (Figure 2c). Generally, in malnourished individuals, homocysteine levels increase significantly, which can cause thrombosis and increase the risk of ischemic heart disease and strokes [40]. Hyperhomocysteinemia is also present in folic acid deficiency and renal failure [41]. In the present study, there is variability in the levels of hemoglobin concentration among different nutritional statuses. The lowest levels of hemoglobin concentration were found in the malnourished group, while the median levels increased as nutritional status improved (Figure 2d). A similar trend was observed in the median hematocrit levels, with significantly higher values in the adequately nourished group (Figure 2e). Magnesium levels also slightly increased with improved nutritional status (Figure 2f). In contrast, calcium levels were significantly lower in the malnourished group than in those at risk of malnutrition (Figure 2g). However, calcium levels were also increased among those with normal nutritional status relative to those with malnutrition. The medians of C-reactive protein levels were similar across all groups. However, the bifurcation values suggested the presence of more subjects with higher CRP levels in the malnourished group (Figure 2h).

### 4.4. Venous Blood Versus Nutritional Status Based on the GNRI

The current study, a comprehensive examination of changes in biochemical and hematological parameters related to the GNRI in the studied senile population, yields significant findings. These findings not only confirm the abovementioned differences between the groups identified by the MNA but also open up new avenues for future research. The study found that the GNRI was similar to the MNA questionnaire in determining the risk of malnutrition based on the amount of total protein (Figure 3a). In addition, there was an increase in albumin concentration, which decreased the risk of malnutrition among the patients. Patients with a high risk of nutritional status based on the GNRI had the lowest concentration of albumin in their blood (Figure 3b). The present study also found that homocysteine levels were highest in older inpatients with a high risk of malnutrition and decreased with improvement in nutritional status, consistent with the results obtained from the MNA (Figure 3c). Hemoglobin concentration (Figure 3d) and hematocrit (Figure 3e) also positively correlated with nutritional status. The study found that the concentration of magnesium (Figure 3f) and calcium (Figure 3g) increased in the subjects’ blood with a reduction in the risk of malnutrition in the GNRI-designated groups. The highest levels of these macronutrients were found in the group with no risk of poor nutritional status. In the case of creatinine, divergence at a statistically significant level was shown between the low malnutrition risk group versus the high malnutrition risk group. Interestingly, the lower median in patients at no risk of malnutrition was observed according to the GNRI than in the low-risk malnutrition group. However, analysis of the boxplot (Figure 3h) indicates that the group of patients with normal nutritional status includes those with the highest creatinine levels. There were too few such cases in the study population for the group with no risk of nutritional-related complications to be distinguished by the highest creatinine levels, so determining the exact relationship would require a more extensive study group. Similarly, in the case of folic acid, determining the relationship would require a more significant study group (Figure 3i). C-reactive protein levels were observed to be highest in patients at high risk of nutritional-related complications, where the median is significantly higher than in other groups (Figure 3j), and CRP decreased with improved nutritional status. An increase in triglycerides was associated with a decrease in malnutrition risk. The groups of patients at low risk of malnutrition and no risk, according to the GNRI, revealed higher triglyceride levels than the high and moderate-risk groups (Figure 3k), which the MNA did not significantly confirm.

### 4.5. Utilization of Biochemical and Hematological Blook Parameters in the Diagnosis of Malnutrition in Older Individuals

Biochemical and hematological venous blood parameters, such as albumin, are commonly used to diagnose malnutrition in patients. However, it is important to note that no evidence-based guidelines fully support their use in specific patient groups and conditions [42]. While it is agreed that laboratory markers should not be relied upon alone but can be used to supplement screening questionnaires and physical examinations, it is crucial to consider the potential misclassification of well-nourished patients [43]. In scientific reports, the concentrations of albumin, hemoglobin, total cholesterol, prealbumin, and total protein were found to be lower in malnourished individuals than in those without malnutrition risk. A meta-analysis performed by Zhang et al. confirmed these markers’ role as malnutrition indicators [39]. The authors simultaneously pointed to the position of CRP, total lymphocyte count, and white blood cells only as indicators of inflammation. At the same time, other biomarkers did not show sufficient evidence to be considered as biomarkers. It confirms the trends observed in the present study, which verified the diagnostic value for malnutrition screening of the most frequently studied parameters during a comprehensive geriatric assessment. Total protein levels in the blood can be used to indicate malnutrition in older individuals. Normally, total protein concentration ranges between 6.0 and 8.3 g/dL. However, according to the abovementioned systematic review and in the present study, using a serum total protein level of less than 6 g/dL as a cutoff point for malnutrition would fail to identify some malnourished individuals who are at nutritional risk according to the MNA and the GNRI. The serum albumin concentration is also a helpful indicator of overall nutritional status in non-acute conditions [44]. However, using a cutoff point of 3.5 g/dL for serum albumin as an indicator of malnutrition may not be appropriate for older individuals, particularly those who are hospitalized. Hypoalbuminemia, defined as a serum albumin concentration of less than 3.5 g/dL, is often considered a standard indicator of malnutrition. Nevertheless, using this value as a cutoff point would result in the under-recognition of malnutrition as defined by commonly used nutritional screening tools [39]. In the current study, this would lead to a failure in the identification of some patients at moderate and low risk of malnutrition, according to the GNRI, and some malnourished patients, according to the MNA. However, in the present study, a serum albumin concentration below 3.0 g/dL occurred in most patients diagnosed with severe malnutrition. Hyperhomocysteinemia, which is typically found in older patients, can increase the risk of vascular disease. A study by Chen et al. conducted in Shanghai on 142 older patients found that blood homocysteine levels were negatively correlated with patients’ nutritional status [45]. Notably, the estimated hemoglobin level in the population studied in this paper was relatively low, even among those with normal nutritional status (mean 12.31 g/dL). This finding supports using a cutoff value of <13 g/dL as an indicator of malnutrition, which is consistent with the lower limit of normal hemoglobin levels for adults according to the WHO (13 g/dL in men and 12 g/dL in women) [46]. The nutritional status of senile patients with a hematocrit level below 36–48% may indicate abnormality [47]. The present study’s mean hematocrit level was low at 35.97% due to low hemoglobin levels. Hematocrit alone cannot be used to diagnose malnutrition in the studied population. Patients who were malnourished or at risk of malnutrition, according to the MNA, and all malnutrition risk groups, according to the GNRI, included individuals who would be undiagnosed based on hematocrit alone. Magnesium, calcium, and folic acid deficiencies indicate qualitative malnutrition in patients. However, there are no reports on their value in assessing the nutritional status of older patients. Magnesium is not among the macronutrients lacking in the daily diet, meaning magnesium deficiency usually results from insufficient food intake [48]. Older individuals are particularly prone to calcium deficiency, which leads to negative calcium balance and loss of bone mass [49]. A previous study involving 84 nursing home patients found that 34% were at risk of malnutrition. However, only 5.9% of the residents had a deficiency of folic acid, which suggests that this deficiency is not very common among the senile population [50]. Scientific reports indicate that creatinine levels may serve as a marker of malnutrition in older dialysis patients [51]. However, the current study conducted on non-dialysis patients and analysis of relevant literature has revealed that creatinine is not a reliable marker of malnutrition in older individuals. Due to the increasing understanding of the role of inflammation in malnutrition, the role of inflammatory mediators as markers of malnutrition is also being investigated. One such mediator is C-reactive protein [43]. In the current study, the median CRP levels in patients at high risk of malnutrition according to the GNRI were significantly higher than in other groups. However, the group of well-nourished patients included those with the highest CRP levels, which would result in them being misclassified as malnourished based solely on C-reactive protein levels. It is important to note that CRP levels are slightly elevated in about one-third of the American population [52]. Additionally, non-nutritional factors such as cardiovascular disease and other conditions of inflammatory origin, like infections, can also affect CRP levels. Therefore, C-reactive protein levels can only serve as an auxiliary marker in assessing nutritional status, particularly in identifying malnutrition caused by inflammation.

Aging is associated with significantly reducing endogenous melatonin secretion, resulting in increased oxidative stress and other metabolic changes [53]. Melatonin is a compound secreted by pinealocytes in a circadian rhythm. Impaired secretion of the hormone can lead to a disruption of the diurnal rhythm of the secretion of adipokines associated with satiety and hunger, such as leptin and ghrelin, leading to excessive energy supply. There are few results in the literature on the effect of melatonin supplementation on weight normalization [54], while there is no information on the role of melatonin in malnutrition in seniors. Also, in the present study, no statistically significant association between the nutritional status of hospitalized patients and the melatonin levels was observed.

IL-6, along with interleukin 1β and TNF-α, is one of the main factors in inflammation associated with the aging process, known as inflammaging [55]. In the physiological acute phase response, IL-6 modulates the synthesis of acute phase factors, including CRP, and enhances immune cell activation. Aging is associated with altered IL-6 trans-signaling, as well as a decrease in the soluble form of IL-6 receptor (sIL-6R) and IL-6 inhibitor (sgp130). Increased signaling due to decreased inhibition induces abnormal activation of cellular IL-6 receptors, exacerbating the inflammatory cascade regardless of antigenic stimuli or tissue damage. The role of nutrition in these processes is of paramount importance. Chronic low-grade inflammation is a significant determinant of anorexia of aging, while acute inflammation can contribute to increased energy requirements, thereby giving rise to disease-related malnutrition. In the present study, a significant increase in the tested serum IL-6 levels was observed as the nutritional status of the patients worsened, as shown by both the boxplots (Figure 2i and Figure 3l) and the medians obtained in each of the study groups. However, malnutrition cannot be diagnosed based on IL-6 levels alone, as some patients with normal nutritional status according to the MNA or at no risk of nutritional-related complications according to the GNRI also had high IL-6 levels. However, according to the results obtained here, a concentration of IL-6 exceeding 7.5 pg/mL may indicate possible nutritional problems. The PolSenior study, a comprehensive investigation involving 4482 patients aged 65 and older, has shed significant light on the predictive role of IL-6 and CRP in physical and cognitive performance, as well as mortality risk in the elderly population. This study, which encompassed subjects with multiple conditions/disorders and those experiencing positive aging, revealed that the lowest IL-6 concentrations were found in normal-weight and overweight individuals, while those with a BMI of less than 18.5 kg/m^2^ and malnourished according to the MNA-SF questionnaire exhibited the highest IL-6 levels. This finding suggests a potential cumulative effect of immune system aging and malnutrition [56].

IP-10 plays a key role in inflammatory diseases. It regulates immune responses by activating and recruiting leukocytes, including T cells, eosinophils, monocytes, and NK cells, through binding to the chemokine receptor CXCR3 [27]. IP-10 is secreted by various cells, including monocytes, neutrophils, endothelial cells, keratinocytes, fibroblasts, mesenchymal cells, dendritic cells, astrocytes, and hepatocytes. Circulating IP-10 levels have been shown to increase in the early stages of type one diabetes. Recent studies have revealed that intrahepatic and circulating IP-10 is associated with obesity and insulin resistance in patients with chronic hepatitis C virus (HCV) infection and in patients with HCV/HIV co-infection. The results of previous studies on IP-10 levels in type 2 diabetes remain inconclusive, and further studies are needed to determine the clinical significance of this chemokine. The increase in serum IP-10 levels observed during the current study, along with the deterioration of the nutritional status as expressed by the MNA score, may be related to malnutrition, with chronic inflammation at its root (Figure 2j) and IP-10 levels above 250 pg/mL may indicate poor nutritional status of the studied senile inpatients.

### 4.6. Limitations of the Study

Despite demonstrating numerous correlations of the studied parameters with the nutritional status of older hospitalized patients, the conducted cross-sectional study has limitations due to the adopted research model, which may be relevant to the conducted inference. The statistical analysis indicates the need to repeat the study in a larger group of patients to establish accurate trends for some biomarkers that could not be established in the group of 137 recruited patients and to specify the sensitivity and specificity of cutoff points. Nevertheless, for several of the parameters determined, differences were identified at a statistically significant level, which sets the direction for future research. It would also be a suitable procedure to analyze the study population, in which the comparison groups would be equal in terms of gender. Despite the similar ages of the different groups determined by the MNA, fewer men participated. Moreover, the analyzed groups were not equal. Also, due to the small size of the group of patients diagnosed with malnutrition according to GLIM criteria, the severity of diagnosed malnutrition was not considered during statistical analysis.

## 5. Conclusions

The present cross-sectional study not only revealed a large scale of the problem of malnutrition in older hospitalized patients in Poland but also showed relationships between the nutritional status and biochemical, hematological, and immunological venous blood parameters routinely tested during the hospital stay. The nutritional status of patients was assessed based on the MNA questionnaire and the GNRI, for which a strong relationship was found. It confirms that both tools determine the nutritional status of older adults in a similar way. The proper diagnosis was made based on the results of screening and the GLIM phenotypic and etiologic criteria. During the hospitalization, selected parameters of venous blood were determined, and the data obtained were compared between different groups based on the MNA and the GNRI. The study confirmed the statistically significant differences between the groups for total protein, albumin, homocysteine, hemoglobin, hematocrit, total magnesium, total calcium, C-reactive protein, IL-6, and IP-10. However, additional significant differences for creatinine, folic acid, and triglycerides were achieved only in the case of the GNRI compartmentalization. According to the results, decreased levels of albumin (<3 g/dL) and hemoglobin (<11 g/dL), elevated homocysteine, CRP, IL-6 (>7.5 pg/mL), and IP-10 (>250 pg/mL) may attract medical processionals’ attention when diagnosing malnutrition. Increased IL-6 and IP-10 levels are presumably associated with inflammaging, while no statistically significant association between the nutritional status of hospitalized seniors and the melatonin levels was identified. It is essential to diagnose malnutrition in every older patient admitted to the hospital, including overweight and obese subjects. This study reiterates the importance of routinely analyzing venous blood parameters to facilitate the early identification of malnutrition and appropriate nutritional management using a specialized diet, engaging medical professionals in the commitment to this practice.

## Figures and Tables

**Table 1:** Distribution of subjects by gender and age by group according to the MNA nutritional status assessment tool.

Study Group	Women		Men		Total N *	Mean Age (±SD **)
	N *	%	N	%		
malnutrition	31	23	16	12	47	82.36 ± 7.40
at risk of malnutrition	9	6	3	2	12	82.68 ± 6.53
normal nutritional status	56	41	22	16	78	79.18 ± 7.96
total	96	70	41	30	137	80.50 ± 7.78

* N—number of patients, ** SD—standard deviation.

**Table 2:** Pearson correlation test results. The means and standard deviation values of the two groups, the value of the correlation coefficient r (X,Y), the coefficient of determination (r^2^), the result of the T (*t*) test, and the probability level of the test (*p*).

Assessment Tool	Mean	Std. Dev.	r (X,Y)	r^2^	*t*	*p*
GNRI value	103.6114	15.03158	0.786652	0.618821	14.80420	<0.000001
MNA score	21.3102	5.27485				

**Table 3:** Mean values of tested parameters, Kruskal–Wallis test results, and *p*-values for selected parameters in the intergroup comparison based on the MNA.

Parameter	Malnutrition <17	At Risk of Malnutrition 17–23.5	Normal Nutritional Status >23.5	Mean	Kruskal–Wallis Test	*p*
total protein	5.79 ± 0.43	6.17 ± 0.38	6.17 ± 0.42	6.03 ± 0.45	H (N = 137) = 21.42589	<0.0001
albumin	3.17 ± 0.48	3.75 ± 0.76	3.71 ± 0.40	3.53 ± 0.53	H (N = 137) = 34.79678	<0.0001
homocysteine	14.90 ± 10.07	12.57 ± 3.35	11.40 ± 4.82	12.67 ± 7.08	H (N = 117) = 8.421101	0.0148
hemoglobin	11.06 ± 1.44	11.77 ± 1.66	12.31 ± 1.51	11.84 ± 1.60	H (N = 137) = 17.21577	0.0002
hematocrit	34.04 ± 4.61	35.23 ± 5.47	37.24 ± 4.36	35.97 ± 4.75	H (N = 137) = 13.93991	0.0009
total magnesium	0.75 ± 0.09	0.81 ± 0.08	0.79 ± 0.09	0.77 ± 0.09	H (N = 137) = 7.850627	0.0197
total calcium	2.15 ± 0.10	2.26 ± 0.16	2.23 ± 0.13	2.20 ± 0.12	H (N = 137) = 11.15462	0.0038
creatinine	1.05 ± 0.48	0.92 ± 0.20	0.96 ± 0.43	0.99 ± 0.43	H (N = 137) = 1.091255	0.5795
vitamin D3	20.02 ± 12.98	17.95 ± 10.16	21.53 ± 12.18	20.70 ± 12.26	H (N = 134) = 1.781448	0.4104
vitamin B12	332.04 ± 209.02	241.25 ± 83.32	301.74 ± 149.09	306.84 ± 168.93	H (N = 137) = 1.745915	0.4177
folic acid	6.05 ± 4.90	6.43 ± 3.74	6.28 ± 3.71	6.21 ± 4.12	H (N = 136) = 3.101100	0.2121
WBC	7.03 ± 2.77	5.76 ± 1.23	8.50 ± 17.46	7.75 ± 13.27	H (N = 137) = 2.774946	0.2497
C-reactive protein	10.92 ± 13.74	6.45 ± 9.49	5.51 ± 9.98	7.45 ± 11.57	H (N = 137) = 14.27547	0.0026
cholesterol	147.55 ± 36.43	173.92 ± 40.22	167.52 ± 47.90	161.23 ± 44.50	H (N = 137) = 5.240044	0.0728
HDL cholesterol	44.04 ± 12.03	47.67 ± 12.42	47.90 ± 12.56	46.55 ± 12.41	H (N = 137) = 2.768267	0.2505
LDL cholesterol	81.66 ± 28.68	103.75 ± 35.97	96.14 ± 41.51	91.84 ± 37.65	H (N = 137) = 4.789838	0.0912
triglycerides	89.85 ± 35.93	96.67 ± 40.56	114.59 ± 61.75	104.53 ± 53.54	H (N = 137) = 5.826718	0.0543
melatonin	85.23 ± 38.32	100.20 ± 63.63	89.57 ± 38.78	89.04 ± 41.15	H (N = 136) = 0.3805290	0.8267
IL-6	12.83 ± 13.76	6.29 ± 10.89	4.41 ± 8.15	7.46 ± 11.26	H (N = 137) = 23.13632	<0.0001
IP-10	277.36 ± 134.23	244.64 ± 107.86	208.59 ± 94.13	236.75 ± 114.81	H (N = 136) = 8.253106	0.0161

Variables that showed significant differences between the study groups are highlighted in blue.

**Table 4:** Mean values of tested parameters, Kruskal–Wallis test results, and *p*-values for selected parameters in the intergroup comparison based on the GNRI.

Parameter	Major Risk of Complications	Moderate Risk of Complications	Low Risk of Complications	No Risk of Complications	Mean	Kruskal–Wallis test	*p*
total protein	5.56 ± 0.41	5.71 ± 0.35	6.00 ± 0.46	6.16 ± 0.40	6.03 ± 0.45	H (3. N = 137) = 28.76349	<0.0001
albumin	2.55 ± 0.35	3.23 ± 0.27	3.40 ± 0.24	3.75 ± 0.45	3.52 ± 0.53	H (3. N = 137) = 60.46830	<0.0001
homocysteine	20.25 ± 17.84	12.70 ± 4.52	13.61 ± 4.55	11.49 ± 4.69	12.67 ± 7.08	H (3. N = 117) = 10.65013	0.0138
hemoglobin	10.29 ± 1.53	11.42 ± 1.27	11.46 ± 1.70	12.21 ± 1.49	11.83 ± 1.60	H (3. N = 137) = 17.31153	0.0006
hematocrit	33.04 ± 6.52	34.34 ± 3.11	35.03 ± 5.32	36.91 ± 4.37	35.96 ± 4.75	H (3. N = 137) = 12.17938	0.0068
total magnesium	0.70 ± 0.08	0.73 ± 0.08	0.78 ± 0.08	0.79 ± 0.09	0.77 ± 0.09	H (3. N = 137) = 17.42125	0.0006
total calcium	2.12 ± 0.11	2.13 ± 0.10	2.19 ± 0.12	2.23 ± 0.12	2.20 ± 0.12	H (3. N = 137) = 15.82245	0.0012
creatinine	0.90 ± 0.54	2.12 ± 0.46	1.18 ± 0.38	0.96 ± 0.42	0.98 ± 0.43	H (3. N = 137) = 15.07416	0.0018
vitamin D3	19.97 ± 14.96	17.54 ± 13.74	20.19 ± 9.02	21.54 ± 12.31	20.70 ± 12.26	H (3. N = 134) = 3.551245	0.3142
vitamin B12	403.50 ± 260.45	241.64 ± 119.93	330.71 ± 210.99	300.48 ± 145.26	306.84 ± 168.93	H (3. N = 137) = 7.261285	0.0640
folic acid	5.32 ± 5.57	4.45 ± 2.87	7.32 ± 4.82	6.42 ± 3.88	6.21 ± 4.12	H (3. N = 136) = 12.31087	0.0064
WBC	6.98 ± 2.22	7.22 ± 1.50	6.68 ± 3.64	8.22 ± 16.55	7.75 ± 13.27	H (3. N = 137) = 5.861460	0.1186
C-reactive protein	20.18 ± 16.20	9.90 ± 11.40	6.68 ± 10.90	5.39 ± 9.91	7.45 ± 11.57	H (3. N = 137) = 14.27547	0.0026
cholesterol	133.67 ± 40.04	150.23 ± 36.18	157.57 ± 36.74	168.06 ± 46.79	161.23 ± 44.50	H (3. N = 137) = 6.050998	0.1092
HDL cholesterol	40.00 ± 10.98	47.00 ± 13.50	44.14 ± 11.38	47.95 ± 12.44	46.55 ± 12.41	H (3. N = 137) = 4.153178	0.2454
LDL cholesterol	70.75 ± 29.86	84.82 ± 28.39	89.80 ± 33.94	96.60 ± 40.18	91.84 ± 37.65	H (3. N = 137) = 5.217342	0.1566
triglycerides	76.42 ± 29.65	86.94±	103.04 ± 41.44	112.20 ± 59.58	104.53 ± 53.54	H (3. N = 137) = 7.849580	0.0492
melatonin	92.34 ± 31.45	75.27 ± 46.87	87.04 ± 32.06	91.78 ± 43.10	89.04 ± 41.15	H (N = 136) = 4.823863	0.1852
IL-6	15.77 ± 13.02	13.90 ± 12.21	8.90 ± 14.32	4.66 ± 8.69	7.46 ± 11.26	H (N = 137) = 31.75766	>0.0001
IP-10	288.70 ± 119.23	249.73 ± 111.90	281.65 ± 163.84	215.97 ± 95.26	236.75 ± 114.81	H (N = 136) = 5.585214	0.1336

Variables that showed significant differences between the study groups are highlighted in blue.

## Data Availability

Data are available on request due to privacy/ethical restrictions.

## Abbreviations

The following abbreviations are used in this manuscript:

MNA	Mini Nutritional Assessment
GNRI	Geriatric Nutrition Risk Index
CRP	C-reactive protein
IL-6	Interleukin 6
IP-10	Interferon γ-induced protein 10
GLIM	Global Leadership Initiative on Malnutrition
CGA	Comprehensive Geriatric Assessment
BMI	Body Mass Index
MNA-SF	Short version of the Mini Nutritional Assessment
WBC	White blood cells
HDL	High-density lipoprotein
LDL	Low-density lipoprotein
Hb	Hemoglobin
HCT	Hematocrit
SGA	Subjective Global Assessment
WHO	World Health Organization
TNF-α	Tumor necrosis factor α
ELISA	Enzyme-Linked Immunosorbent Assay

## References

[1] Badrkhahan S.Z., Ala M., Fakhrzadeh H., Yaghoobi A., Mirzamohamadi S., Arzaghi S.M., Shahabi S., Sharifi F., Ostovar A., Fahimfar N. (2023). The Prevalence and Predictors of Geriatric Giants in Community-Dwelling Older Adults: A Cross-Sectional Study from the Middle East. Sci. Rep..

[2] Volkert D. (2013). Malnutrition in Older Adults-Urgent Need for Action: A Plea for Improving the Nutritional Situation of Older Adults. Gerontology.

[3] Malenfant J.H., Batsis J.A. (2019). Obesity in the Geriatric Population—A Global Health Perspective. J. Glob. Health Rep..

[4] Özkaya I., Gürbüz M. (2019). Malnourishment in the Overweight and Obese Elderly. Nutr. Hosp..

[5] Krzymińska-Szymaszko R., Lewandowicz M., Wieczorowska-Tobis K. (2016). Niedożywienie Jako Wielki Zespół Geriatryczny Malnutrition as a Geriatric Giant. Geriatria.

[6] MNA® as Part of the Comprehensive Geriatric Assessment (CGA). https://www.mna-elderly.com/geriatric-assessment.

[7] DiMnrin-Ghnlili R.A. (2014). Integrating Nutrition in the Comprehensive Geriatric Assessment. Nutr. Clin. Pract..

[8] Mziray M., Zuralska R., Ksiazek J., Domagała P. (2016). Niedożywienie u Osób w Wieku Podeszłym, Metody Jego Oceny, Profilaktyka i Leczenie. Ann. Acad. Medicae Gedanensis.

[9] Sobotka L. (2012). Basics in Clinical Nutrition.

[10] Cederholm T., Jensen G.L., Correia M.I.T.D., Gonzalez M.C., Fukushima R., Higashiguchi T., Baptista G., Barazzoni R., Blaauw R., Coats A. (2019). GLIM Criteria for the Diagnosis of Malnutrition—A Consensus Report from the Global Clinical Nutrition Community. Clin. Nutr..

[11] Raynaud-Simon A. (2009). Virtual Clinical Nutrition University: Malnutrition in the Elderly, Epidemiology and Consequences. e-SPEN.

[12] Burks C.E., Jones C.W., Braz V.A., Swor R.A., Richmond N.L., Hwang K.S., Hollowell A.G., Weaver M.A., Platts-Mills T.F. (2017). Risk Factors for Malnutrition among Older Adults in the Emergency Department: A Multicenter Study. J. Am. Geriatr. Soc..

[13] Adams N.E., Bowie A.J., Simmance N., Murray M., Crowe T.C. (2008). Recognition by Medical and Nursing Professionals of Malnutrition and Risk of Malnutrition in Elderly Hospitalised Patients. Nutr. Diet..

[14] Kaur B., Mal H.K. (2017). Association between Malnutrition and Depression among Elderly of Selected Rural Area of District Faridkot, Punjab. Int. J. Community Health Med. Res..

[15] Klimek E., Parnicka A. Żywienie W Geriatrii I Opiece Długoterminowej—Zagadnienia Ogólne. https://www.mp.pl/geriatria/wytyczne/129325,zywienie-w-geriatrii-i-opiece-dlugoterminowej-zagadnienia-ogolne?fbclid=IwAR2QmP1e3xFANy468n22mGnWA4Ul10l5FpGuNZyxc4W2yq-VwhtiaqEHA7w.

[16] Kondrup J., Allison S.P., Elia M., Vellas B., Plauth M. (2003). ESPEN Guidelines for Nutrition Screening 2002. Clin. Nutr..

[17] Vellas B., Guigoz Y., Garry P.J., Nourhashemi F., Bennahum D., Lauque S., Albarede J.L., Vellas B. (1999). The Mini Nutritional Assessment (MNA) for Grading the Nutritional State of Elderly Patients: Presentation of the MNA, History and Validation. Nestle Nutr. Workshop Ser. Clin. Perform. Programme.

[18] Donini L.M., Poggiogalle E., Molfino A., Rosano A., Lenzi A., Rossi Fanelli F., Muscaritoli M. (2016). Mini-Nutritional Assessment, Malnutrition Universal Screening Tool, and Nutrition Risk Screening Tool for the Nutritional Evaluation of Older Nursing Home Residents. J. Am. Med. Dir. Assoc..

[19] Kuzuya M., Kanda S., Koike T., Suzuki Y., Iguchi A. (2005). Lack of Correlation between Total Lymphocyte Count and Nutritional Status in the Elderly. Clin. Nutr..

[20] Abd Aziz N.A.S., Mohd Fahmi Teng N.I., Kamarul Zaman M. (2019). Geriatric Nutrition Risk Index Is Comparable to the Mini Nutritional Assessment for Assessing Nutritional Status in Elderly Hospitalized Patients. Clin. Nutr. ESPEN.

[21] Gärtner S., Kraft M., Krüger J., Vogt L.J., Fiene M., Mayerle J., Aghdassi A.A., Steveling A., Völzke H., Baumeister S.E. (2017). Geriatric Nutritional Risk Index Correlates with Length of Hospital Stay and Inflammatory Markers in Older Inpatients. Clin. Nutr..

[22] Durán Alert P., Milà Villarroel R., Formiga F., Virgili Casas N., Vilarasau Farré C. (2012). Evaluación de Los Métodos de Cribaje de Riesgo Nutricional En Pacientes Geriátricos; Mini Nutritional Assessment (MNA) versus Geriatric Nutritional Risk Assessment (GNRI). Nutr. Hosp..

[23] Sampaio L.R. (2004). Avaliação Nutricional e Envelhecimento. Rev. Nutr..

[24] Cederholm T., Bosaeus I., Barazzoni R., Bauer J., Van Gossum A., Klek S., Muscaritoli M., Nyulasi I., Ockenga J., Schneider S.M. (2015). Diagnostic Criteria for Malnutrition—An ESPEN Consensus Statement. Clin. Nutr..

[25] Bobin-Dubigeon C., Lefrançois A., Vansteene D., Dupé M., Joalland M.P., Bard J.M. (2017). Leptin and Adiponectin as New Markers of Undernutrition in Cancer. Clin. Biochem..

[26] Rea I.M., Gibson D.S., McGilligan V., McNerlan S.E., Denis Alexander H., Ross O.A. (2018). Age and Age-Related Diseases: Role of Inflammation Triggers and Cytokines. Front. Immunol..

[27] Chang C.-C., Wu C.-L., Su W.-W., Shih K.-L., Tarng D.-C., Chou C.-T., Chen T.-Y., Kor C.-T., Wu H.-M. (2015). Interferon Gamma-Induced Protein 10 Is Associated with Insulin Resistance and Incident Diabetes in Patients with Nonalcoholic Fatty Liver Disease. Sci. Rep..

[28] Hernández-Trejo M., Montoya-Estrada A., Torres-Ramos Y., Espejel-Núñez A., Guzmán-Grenfell A., Morales-Hernández R., Tolentino-Dolores M., Laresgoiti-Servitje E. (2017). Oxidative Stress Biomarkers and Their Relationship with Cytokine Concentrations in Overweight/Obese Pregnant Women and Their Neonates. BMC Immunol..

[29] Szewczyk-Golec K., Woźniak A., Reiter R.J. (2015). Inter-Relationships of the Chronobiotic, Melatonin, with Leptin and Adiponectin: Implications for Obesity. J. Pineal Res..

[30] Krzymińska-Siemaszko R., Mossakowska M., Skalska A., Klich-Rączka A., Tobis S., Szybalska A., Cylkowska-Nowak M., Olszanecka-Glinianowicz M., Chudek J., Wieczorowska-Tobis K. (2015). Social and Economic Correlates of Malnutrition in Polish Elderly Population: The Results of PolSenior Study. J. Nutr. Heal. Aging.

[31] Cereda E., Pedrolli C., Klersy C., Bonardi C., Quarleri L., Cappello S., Turri A., Rondanelli M., Caccialanza R. (2016). Nutritional Status in Older Persons According to Healthcare Setting: A Systematic Review and Meta-Analysis of Prevalence Data Using MNA^®^. Clin. Nutr..

[32] Bouillanne O., Morineau G., Dupont C., Coulombel I., Vincent J.-P., Nicolis I., Benazeth S., Cynober L., Aussel C. (2005). Geriatric Nutritional Risk Index: A New Index for Evaluating at-Risk Elderly Medical Patients. Am. J. Clin. Nutr..

[33] Orrevall Y., Tishelman C., Permert J., Cederholm T. (2009). Nutritional Support and Risk Status among Cancer Patients in Palliative Home Care Services. Support. Care Cancer.

[34] Moreira N.C.F., Krausch-Hofmann S., Matthys C., Vereecken C., Vanhauwaert E., Declercq A., Bekkering G.E., Duyck J. (2016). Risk Factors for Malnutrition in Older Adults: A Systematic Review of the Literature Based on Longitudinal Data. Adv. Nutr..

[35] Nazan S., Buket K. (2018). Evaluation of Nutritional Status of Elderly Patients Presenting to the Family Health Center. Pak. J. Med. Sci..

[36] Alzahrani S.H., Alamri S.H. (2017). Prevalence of Malnutrition and Associated Factors among Hospitalized Elderly Patients in King Abdulaziz University Hospital, Jeddah, Saudi Arabia. BMC Geriatr..

[37] Luo Y., Rahmati M., Kazemi A., Liu W., Lee S.W., Gyasi R.M., López Sánchez G.F., Koyanagi A., Smith L., Yon D.K. (2024). Effects of Therapeutic Ultrasound in Patients with Knee Osteoarthritis: A Systematic Review and Meta-Analysis. Heliyon.

[38] Rahmati M., Lee S., Yon D.K., Lee S.W., Udeh R., McEvoy M., Oh H., Butler L., Keyes H., Barnett Y. (2024). Physical Activity and Prevention of Mental Health Complications: An Umbrella Review. Neurosci. Biobehav. Rev..

[39] Zhang Z., Pereira S.L., Luo M., Matheson E.M. (2017). Evaluation of Blood Biomarkers Associated with Risk of Malnutrition in Older Adults: A Systematic Review and Meta-Analysis. Nutrients.

[40] Clarke R., Collins R., Lewington S., Donald A., Alfthan G., Tuomilehto J., Arnesen E., Bonaa K., Blacher J., Boers G.H.J. (2002). Homocysteine and Risk of Ischemic Heart Disease and Stroke: A Meta-Analysis. J. Am. Med. Assoc..

[41] Devalia V., Hamilton M.S., Molloy A.M. (2014). Guidelines for the Diagnosis and Treatment of Cobalamin and Folate Disorders. Br. J. Haematol..

[42] Raiten D.J., Brabin B., Combs G., Abbe M.R.L., Wasantwisut E., Namaste S., Darnton-hill I. (2011). Executive Summary—Biomarkers of Nutrition for Development: Building a Consensus. Am. J. Clin. Nutr..

[43] Bharadwaj S., Ginoya S., Tandon P., Gohel T.D., Guirguis J., Vallabh H., Jevenn A., Hanouneh I. (2016). Malnutrition: Laboratory Markers vs Nutritional Assessment. Gastroenterol. Rep..

[44] Marín-Ciancas F., Malafarina V., Cuadras D., Cabrerizo S., Gomez-Busto F., Artaza-Artabe I. (2015). Serum Albumin and Health in Older People: Review and Meta Analysis. Maturitas.

[45] Chen S.F., Cui C.L., Wu P., Xie N.Z. (2013). Relationship of Serum Homocysteine Level with Nutritional Status and HbA1c Level in Elderly Inpatients. Int. J. Clin. Exp. Med..

[46] WHO (1968). Scientific Group WHO Technical Report on Nutritional Anaemias.

[47] De São José J.F.B., Ribeiro A.Q., De Oliveira F.C.E., Gomide C.I., Alfenas R.d.C.G., Paula H.A.d.A. (2016). Nutritional Screening of Elderly by Different Methods and Indicators Asmitted to Hospital. DEMETRA Aliment. Nutr. Saúde.

[48] Sakaguchi Y., Fujii N., Shoji T., Hayashi T., Rakugi H., Isaka Y. (2014). Hypomagnesemia Is a Significant Predictor of Cardiovascular and Non-Cardiovascular Mortality in Patients Undergoing Hemodialysis. Kidney Int..

[49] Iuliano S., Poon S., Robbins J., Bui M., Wang X., De Groot L., Van Loan M., Zadeh A.G., Nguyen T., Seeman E. (2021). Effect of Dietary Sources of Calcium and Protein on Hip Fractures and Falls in Older Adults in Residential Care: Cluster Randomised Controlled Trial. BMJ.

[50] Brito Noronha M., Cunha N.A., Araújo D.A., Flamínio Abrunhosa S., Rocha A.N., Amaral T.F. (2015). Undernutrition, Serum Vitamin B12, Folic Acid and Depressive Symptoms in Older Adults. Nutr. Hosp..

[51] Yildiz A., Tufan F. (2015). Lower Creatinine as a Marker of Malnutrition and Lower Muscle Mass in Hemodialysis Patients. Clin. Interv. Aging.

[52] Kushner I., Rzewnicki D., Samols D. (2006). What Does Minor Elevation of C-Reactive Protein Signify?. Am. J. Med..

[53] Liguori I., Russo G., Curcio F., Bulli G., Aran L., Della-Morte D., Gargiulo G., Testa G., Cacciatore F., Bonaduce D. (2018). Oxidative Stress, Aging, and Diseases. Clin. Interv. Aging.

[54] Nuszkiewicz J., Kwiatkowska A., Majko K., Wesołowski R., Szewczyk-Golec K. (2017). Stres Oksydacyjny i Stan Zapalny a Rozwój Otyłości: Protekcyjne Działanie Melatoniny. Probl. Hig. i Epidemiol..

[55] Ticinesi A., Meschi T., Lauretani F., Felis G., Franchi F., Pedrolli C., Barichella M., Benati G., Di Nuzzo S., Ceda G.P. (2016). Nutrition and Inflammation in Older Individuals: Focus on Vitamin D, n-3 Polyunsaturated Fatty Acids and Whey Proteins. Nutrients.

[56] Puzianowska-Kuźnicka M., Owczarz M., Wieczorowska-Tobis K., Nadrowski P., Chudek J., Slusarczyk P., Skalska A., Jonas M., Franek E., Mossakowska M. (2016). Interleukin-6 and C-Reactive Protein, Successful Aging, and Mortality: The PolSenior Study. Immun. Ageing.

